# Adaptive limited feedback for interference alignment in MIMO interference channels

**DOI:** 10.1186/s13638-016-0724-z

**Published:** 2016-09-20

**Authors:** Yang Zhang, Chenglin Zhao, Juan Meng, Shibao Li, Li Li

**Affiliations:** 1 School of Information and Communication Engineering, BeiJing University of Posts and Telecommunications, Xitucheng Road, Beijing, 100876 China; 2 College of Computer and Communication Engineering, China University of Petroleum, Changjiang West Road, Qingdao, 266580 China; 3 Department of Disaster Prevention Instrument, Institute of Disaster Prevention, Xueyuan Road, Sanhe, 065201 China

**Keywords:** MIMO, Interference alignment, Limited feedback, Waterfilling

## Abstract

It is very important that the radar sensor network has autonomous capabilities such as self-managing, etc. Quite often, MIMO interference channels are applied to radar sensor networks, and for self-managing purpose, interference management in MIMO interference channels is critical. Interference alignment (IA) has the potential to dramatically improve system throughput by effectively mitigating interference in multi-user networks at high signal-to-noise (SNR). However, the implementation of IA predominantly relays on perfect and global channel state information (CSI) at all transceivers. A large amount of CSI has to be fed back to all transmitters, resulting in a proliferation of feedback bits. Thus, IA with limited feedback has been introduced to reduce the sum feedback overhead. In this paper, by exploiting the advantage of heterogeneous path loss, we first investigate the throughput of IA with limited feedback in interference channels while each user transmits multi-streams simultaneously, then we get the upper bound of sum rate in terms of the transmit power and feedback bits. Moreover, we propose a dynamic feedback scheme via bit allocation to reduce the throughput loss due to limited feedback. Simulation results demonstrate that the dynamic feedback scheme achieves better performance in terms of sum rate.

## Introduction

The network of radar sensors should operate with multiple goals managed by an intelligent platform network that can manage the dynamics of each radar to meet the common goals of the platform [1, 2], rather than each radar to operate as an independent system. Therefore, it is significant to perform signal design and processing, and networking cooperatively within and between platforms of radar sensors and their communication modules [3]. In addition, radar sensor network’s performance can be greatly enhanced by changing the radar’s algorithms as the mission changes (mission aware), and it is also very important that the radar sensor network has autonomous capabilities [4, 5]: self-organizing, self-healing, self-reconfiguring, and self-managing [6], etc. Quite often, MIMO interference channels are applied to radar sensor networks [7], and for self-managing purpose, interference management in MIMO interference channels is critical, especially when the channel conditions are not friendly such as foliage environment [8]. In this paper, we study interference alignment in MIMO interference channels.

Interference is one of the key roadblocks to improving the throughput of multi-user wireless networks. By aligning the interference from other users to a lower-dimensional subspace, interference alignment (IA) allocates higher dimensional subspace for desired receivers, and achieves the optimal degrees of freedom (DoF). In *K*-user interference channels, IA is shown to achieve full *K*/2 multiplexing gain per time, frequency and antenna dimension [9]. Encouraged by this surprising result, extensive works have proposed various schemes to align interference at each receiver by novel designed signal structures [10, 11].

For most of IA approaches, perfect CSI is assumed to be available at all transceivers. As is well known, CSI can be obtained at transmitters through the reciprocity between forward and reverse channels in time division duplex (TDD) systems. However, in frequency division duplex (FDD) systems, CSI is sent from receivers to transmitters via feedback links, whose data rate is often severely limited. This issue of IA with limited feedback in MIMO networks is widely researched in the work [12] and its references.

The throughput loss of IA technology due to limited feedback of MISO frequency selected channel is first investigated in [13], and this work is extended to MIMO frequency selected channel in [14]. For an interference channel with *N* antennas per user and the transmit power *P*, the works in [13] and [14] show that each user achieves the optimal DoF while the channel coherence time is large and the feedback rate at least (*N*^2^−1)*log*_2_*P*. The effective DoF achieved by IA is studied in [15] when both channel estimation and CSI limited feedback are taken into consideration. Based on the closed-form solution of transmit precoders for a special interference channel, a novel feedback topology for IA has been designed in [16], and it reduces feedback overhead significantly compared with conventional feedback frameworks.

Grassmannian codebooks are widely used to quantize the CSI [13–15]. It has been well-known that the Grassmannian codebooks are optimal for independent identically distributed channels, but it is challenging to design the optimal codebooks except for some special cases. Therefore, it is impractical to use Grassmannian codebooks for limited feedback [17, 18]. Random vector quantization (RVQ) codebooks, which consist of independent isotopically distributed unit norm vectors, are proposed to reduce the design complexity in [19] and [20]. For moderate size codebooks, RVQ codebooks perform close to the Grassmannian codebooks for wireless channels with independent identically distributed gain. An IA scheme with RVQ-based quantized transmit precoders for MIMO interference channel is proposed in [21].

It is noteworthy that, in previous works, the CSI from different interfering transmitters is quantized with symmetric codebooks. Intuitively, if the interfering links have different path loss, they should not be quantized with the same amount of bits. To exploit the potential advantage of heterogeneous path loss, the works [16] and [22] propose dynamic quantization schemes via bit allocations. They adaptively allocate the number of feedback bits under the constraints of total feedback bits for whole systems. However, these works only investigate systems while each user transmits only single stream simultaneously, and the assumption of all receivers’ cooperation is impractical in downlink networks. In this paper, we extend these investigations from single stream to multi streams for each user without cooperation between receivers. We first evaluate the throughput loss of IA due to limited feedback. On this basis, we optimize the receivers bit allocation scheme to decrease the throughput loss by exploiting the advantage of heterogeneous path loss.

The rest of this paper is organized as follows. In Section 2, the system model is introduced. In Section 3, the throughput loss is analyzed and an optimal bit allocation scheme is addressed. Simulation results are provided in Section 4, and the conclusions are given in the last section.

*Notations:* The superscripts ^*T*^,^*H*^ and ^∗^ denote transposition, Hermitian transpose and element-wise conjugate, respectively. *E*[∙] stands for the expectation operator. Lower-case and upper-case bold-face letters are used to vectors and matrices, respectively. *tr*(**A**) is the trace of the matrix **A**. |**x**| is the absolute value of the complex scalar **x** and ∥**A**∥_*F*_ is the Frobenius norm of the matrix **A**.

## System model

**Fig. 1:**
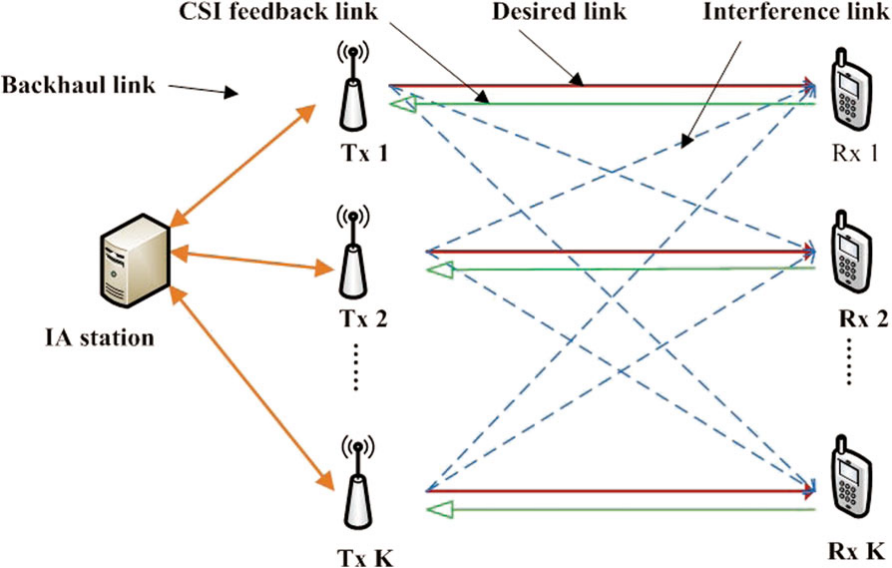
System model for IA with limited feedback

As shown in Fig. 1, we consider a *K*-user MIMO interference channel. For simplicity, we investigate the case of the homogeneous network where each transmitter and receiver is equipped with *N* antennas, respectively. Thus, each transmitter delivers *D*(*D*≤*N*) independent data streams to its corresponding receiver and provided that IA remains feasible.

Considering a block fading channel model, in which channels are drawn independently across all users and antennas, and remain fixed for a constant interval. Under the assumption of perfect CSI at the transmitters, the sampled baseband signal at receiver *k* for the *d*-th data stream can be written as

1 $${} \begin{aligned} \left(\mathbf{u}^{d}_{k}\right)^{H} \mathbf{y}_{k} &=\left(\mathbf{u}^{d}_{k}\right)^{H}\sqrt{\eta_{k,k}}\mathbf{H}_{k,k}\mathbf{v}^{d}_{k}\mathbf{x}^{d}_{k} \\ &\quad+ \left(\mathbf{u}^{d}_{k}\right)^{H} \sum\limits^{D}_{j=1,j \neq d} \sqrt{\eta_{k,k}} \mathbf{H}_{k,k} \mathbf{v}^{j}_{k} \mathbf{x}^{j}_{k} \\ &\quad+ \left(\mathbf{u}^{d}_{k}\right)^{H} \sum\limits^{K}_{i=1,i \neq k} \sum\limits^{D}_{j=1}\sqrt{\eta_{k,i}}\mathbf{H}_{k,i}\mathbf{v}^{j}_{i} \mathbf{x}^{j}_{i} + \left(\mathbf{u}^{d}_{k}\right)^{H} \mathbf{z}_{k} \end{aligned}  $$

Where $\mathbf {x}_{k} = \left [x^{1}_{k},\cdots {x^{D}_{k}}\right ]^{T}$ denotes data symbols, and the symbols in **x**_*k*_ are assumed to be independently generated with unit variance. $\mathbf {z}_{k} \in {\mathbb {C}}^{D \times 1}$ is the additive white Gaussian noise vector observed at receiver *k*. $\mathbf {V}_{i} = \left [\mathbf {v}^{1}_{i}, \cdots \mathbf {v}^{D}_{i} \right ]$ and $\mathbf {U}_{i} = \left [\mathbf {u}^{1}_{i}, \cdots \mathbf {u}^{D}_{i} \right ]$ indicate the precoding and receiving filter matrices, respectively. ∀1≤*k*,*i*≤*K*,1≤*d*,*j*≤*D*, we have $\parallel \mathbf {v}_{i}^{j} \parallel _{\textmd {F}} = \parallel \mathbf {u}_{i}^{j} \parallel _{\textmd {F }}= 1, \eta _{k,i} $ is the path loss of the channel from transmitter *i* to receiver *k*, while **H**_*k*,*i*_ is the small scale fading of channel. Each entity of **H**_*k*,*i*_ are assumed to be independently and identically distributed complex Gaussian random variables with zero mean and unit variance. And, we only pay attention to the feedback of small scale fading **H**_*k*,*i*_. For path loss, we choose a model for outdoor to indoor and pedestrian test environment recommended by Radiocommunication Sector of the International Telecommunication Union (ITU-R). The path loss is given by:

2 $$ \eta_{k,i} = 40 \log_{10} r_{k,i} + 30 \log_{10} f + 49 [\textmd{dB}]  $$

where *r*_*k*,*i*_ is the distance from transmitter *i* to receiver *k*.

With perfect CSI, the precoding vectors should satisfy [2]

3 $$ \left\{\begin{array}{l} (\mathbf{u}_{k}^{d})^{H} \mathbf{H}_{k,i} \mathbf{v}_{i}^{j} = 0, \forall k \neq i \\ (\mathbf{u}_{k}^{d})^{H} \mathbf{H}_{k,k} \mathbf{v}_{k}^{j} = 0, \forall d \neq j \\ \left| (\mathbf{u}_{k}^{d})^{H} \mathbf{H}_{k,k} \mathbf{v}_{k}^{d} \right| > 0, \forall k,d \end{array}\right.  $$

Therefore, the throughput of system with perfect CSI is

4 $$ \begin{aligned} R_{\Sigma} &= \sum\limits_{k=1}^{K} \sum\limits_{d=1}^{D} {R_{k}^{d}}\\ &= \sum\limits_{k=1}^{K} \sum\limits_{d=1}^{D} \log_{2} \left(1 + \frac{\frac{P}{D} \eta_{k,k} \left| (\mathbf{u}_{k}^{d})^{H} \mathbf{H}_{k,k} \mathbf{v}_{k}^{d} \right|^{2}}{\sigma^{2}} \right) \end{aligned}  $$

From above equations, CSI must be acquired and exchanged through channel estimation, quantization and feedback. The details are shown in [10] and [17]; we will not repeat it.

It is noteworthy that, in downlink networks, there is no coordinate node connected with receivers via high capacity backhaul links. At each receiver k, the CSI {**H**_*k*,*i*_} from transmitter *i* to receiver *k* are quantized to be $\{\bar {\mathbf {H}}_{k,i}\}$ using a local codebook. The quantized codeword indexes are then fed back to the transmitter *j* via fixed rate feedback channels. The feedback bit rate derived in this paper targets a periodic feedback protocol, which is more suitable for existing communication standards such as 3GPP-LTE, where a fixed number of bits in each data block of reverse links are allocated for CSI feedback. For simplify, we assume *B*_*f*_ feedback bits per data block to be delivered from each receiver. As illustrated in Fig. 1, we assume that there is an IA station with backhaul connections to all the transmitters. The IA station performs IA schemes based on global CSI collected from transmitters, and then distributes precoding matrices and receiving filter matrices to all transmitters. With the quantized CSI $\{\bar {\mathbf {H}}_{k,i}\}$, the IA station calculates the precoding vectors $\{\bar {\mathbf {v}}_{i}\}$, yielding

5 $$ \left\{\begin{array}{l} (\bar{\mathbf{u}}_{k}^{d})^{H} \bar{\mathbf{H}}_{k,i} \bar{\mathbf{v}}_{i}^{j} = 0, \forall k \neq i \\ (\bar{\mathbf{u}}_{k}^{d})^{H} \bar{\mathbf{H}}_{k,k} \bar{\mathbf{v}}_{k}^{j} = 0, \forall d \neq j \\ \left| (\bar{\mathbf{u}}_{k}^{d})^{H} \bar{\mathbf{H}}_{k,k} \bar{\mathbf{v}}_{k}^{d} \right| > 0, \forall k,d \end{array}\right.  $$

where $\bar {\mathbf {v}}_{i}^{j}$ and $\bar {\mathbf {u}}_{k}^{d}$ are the beamforming and receive filter vectors which designed for quantized channels. Thus, we have

6 $$ \begin{aligned} \left(\bar{\mathbf{u}}^{d}_{k}\right)^{H} \bar{\mathbf{y}}_{k} &=\left(\bar{\mathbf{u}}^{d}_{k}\right)^{H}\sqrt{\eta_{k,k}}\mathbf{H}_{k,k}\bar{\mathbf{v}}^{d}_{k}\mathbf{x}^{d}_{k}\\ &\quad+ \left(\bar{\mathbf{u}}^{d}_{k}\right)^{H} \sum\limits^{D}_{j=1,j \neq d} \sqrt{\eta_{k,k}} \mathbf{H}_{k,k} \bar{\mathbf{v}}^{j}_{k} \mathbf{x}^{j}_{k} \\ &\quad+ \left(\bar{\mathbf{u}}^{d}_{k}\right)^{H} \sum\limits^{K}_{i=1,i \neq k} \sum\limits^{D}_{j=1}\sqrt{\eta_{k,i}}\bar{\mathbf{v}}^{j}_{i} \mathbf{x}^{j}_{i} + \left(\bar{\mathbf{u}}^{d}_{k}\right)^{H} \mathbf{z}_{k} \end{aligned}  $$

The throughput of system employ IA technology with limited feedback is:

7 $$ \begin{aligned} \bar{R}_{\Sigma} &= \sum\limits_{k=1}^{K} \sum\limits_{d=1}^{D} \bar{R}_{k}^{d} \\ &= \sum\limits_{k=1}^{K} \sum\limits_{d=1}^{D} \log_{2} \left (1 + \frac{\frac{P}{D} \eta_{k,k} \left|(\bar{\mathbf{u}}_{k}^{d})^{H} \mathbf{H}_{k,k} \bar{\mathbf{v}}_{k}^{d} \right|^{2}}{\bar{I}_{k,1}^{d} + \bar{I}_{k,2}^{d} + \sigma^{2}} \right) \end{aligned}  $$

where $\bar {I}_{k,1}^{d}$ is the intra-user interference from the transmitter *k*, and is the inter-user interference from other transmitters, as shown as the follows

8 $$ \bar{I}_{k,1}^{d} = \frac{P}{D} \sum\limits_{j = 1,j \neq d}^{D}\eta_{k,k}\left| (\bar{\mathbf{u}}_{k}^{d})^{H} \mathbf{H}_{k,k} \bar{\mathbf{v}}_{k}^{j} \right|^{2}  $$

9 $$ \bar{I}_{k,2}^{d} = \frac{P}{D}\sum\limits_{i=1,i \neq k}^{K} \sum\limits_{j = 1}^{D}\eta_{k,i}\left| (\bar{\mathbf{u}}_{k}^{d})^{H} \mathbf{H}_{k,i} \bar{\mathbf{v}}_{i}^{j} \right|^{2}  $$

## Dynamic feedback bit allocation

To minimize the throughput loss due to limited feedback, dynamic feedback-bit allocation strategies that adaptively distribute the number of feedback bits to each pair of links are introduced in [11] and [17]. However, a centralized bit controller is assumed to gather channel gains from all receivers and computes the number of feedback-bits for each link. It is impractical for downlink networks. In this section, we allocate the feedback-bits among the interfering links of each receiver but not all receivers without receiver cooperation. We first introduce the principle of CSI quantization. Then, we investigate the throughput loss in the feedback topology proposed in the previous section. Finally, we introduce the dynamic feedback bit allocation strategy.

### CSI Quantization

Take the example of receiver *k*, we describe the process of CSI quantization briefly. We denote *B* as the number of feedback bits for **H**_*k*,*i*_. The receiver *k* first expands the CSI **H**_*k*,*i*_ into a vector **h**_*k*,*i*_, which is quantized based on a local codebook $\mathcal {C}$ as

10 $$ \bar{\mathbf{h}}_{k,i} = \arg \min_{\omega_{m} \in \mathcal{C}} \textmd{dist}\left(\mathbf{h}_{k,i}, \omega_{m}\right)  $$

where *ω*_*m*_ is of unit-norm, the size of codebook is $2^{B}, \textmd {dist}(\mathbf {h}_{k,i}, \omega _{m}) = \sqrt {1-|\mathbf {h}_{k,i}^{H} \omega _{m}|^{2}}$ denotes the chordal distance between vectors **h**_*k*,*i*_ and *ω*_*m*_. the quantizing result is the codeword indexe which has the minimize distance between vector **h**_*k*,*i*_ in codebook $\mathcal {C}$. It is found and fed back to the transmitter *k*. As a result, the quantized CSI $\bar {\mathbf {H}}_{k,i}$ is obtained by reshape the codeword which obtained from codebook with the received index.

To characterize the performance loss of the IA with limited feedback, we introduce an upper bound of the throughput loss as a function of the number of feedback bits. Using the quantized CSI $\bar {\mathbf {H}}_{k,i}$, the CSI **H**_*k*,*i*_ can be decomposed as [23]

11 $$ \mathbf{H}_{k,i} = \cos \theta_{k, i}\bar{\mathbf{H}}_{k, i} + \sin \theta_{k, i} \Delta \mathbf{H}_{k, i}  $$

where the principal angles are defined as $\theta _{i} = \arccos \left | \mathbf {H}_{k, i}^{H} \bar {\mathbf {H}}_{k, i} \right |$, and *Δ***H**_*k*,*i*_ is the error vector due to the quantization with $\| \Delta \mathbf {H}_{k, i} \|_{\textmd {F}} = \| \bar {\mathbf {H}}_{k, i} \|_{\textmd {F}} = 1$.

### Throughput loss analysis

For the limited feedback systems, both intra-user interference and inter-user interference cannot be eliminated perfectly so that the residual interference degrades the throughput of systems. We denote the rate loss for the *d*th stream of the *k*th receiver as $\Delta {R_{k}^{d}} = \textmd {E}[{R_{k}^{d}} - \bar {R}_{k}^{d}]$. Then, the throughput loss is upper-bounded as below:

12 $$ \begin{aligned} \Delta {R_{k}^{d}} &= \textmd{E}\left[ \log_{2} \left(1 + \frac{\frac{P}{D}\eta_{k,k} \left| (\mathbf{u}_{k}^{d})^{H} \mathbf{H}_{k,k} \mathbf{v}_{k}^{d} \right|^{2}}{\sigma^{2}} \right) \right]\\ &\quad-\textmd{E} \left[ \log_{2} \left(1 + \frac{\frac{P}{D} \eta_{k,k}\left|(\bar{\mathbf{u}}_{d}^{k})^{H} \mathbf{H}_{k,k} \bar{\mathbf{v}}_{k}^{d} \right|^{2}}{\bar{I}_{k,1}^{d} + \bar{I}_{k,2}^{d} + \sigma^{2}} \right) \right] \\ &\overset{(a)}{=}\textmd{E}\left[ \log_{2} \left(1 + \frac{\frac{P}{D}\eta_{k,k} \left| (\bar{\mathbf{u}}_{k}^{d})^{H} \mathbf{H}_{k,k} \bar{\mathbf{v}}_{k}^{d} \right|^{2}}{\sigma^{2}} \right) \right] \\ &\quad-\textmd{E} \left[ \log_{2} \left(1 + \frac{\frac{P}{D} \eta_{k,k}\left|(\bar{\mathbf{u}}_{d}^{k})^{H} \mathbf{H}_{k,k} \bar{\mathbf{v}}_{k}^{d} \right|^{2}}{\bar{I}_{k,1}^{d} + \bar{I}_{k,2}^{d} + \sigma^{2}} \right) \right]\\ &\overset{(b)}{\leq}\textmd{E}\left[ \log_{2} \left(1 + \frac{P\eta_{k,k}}{D\sigma^{2}}\left| (\bar{\mathbf{u}}_{k}^{d})^{H} \mathbf{H}_{k,k} \bar{\mathbf{v}}_{k}^{d} \right|^{2} \right) \right]\\ &\quad+ \textmd{E}\left[\log_{2} \left(1 + \frac{\bar{I}_{k,1}^{d}}{\sigma^{2}} + \frac{\bar{I}_{k,2}^{d}}{\sigma^{2}} \right)\right]\\ &\quad -\textmd{E}\left[ \log_{2} \left(1 + \frac{P\eta_{k,k}}{D\sigma^{2}} \left| (\bar{\mathbf{u}}_{k}^{d})^{H} \mathbf{H}_{k,k} \bar{\mathbf{v}}_{k}^{d} \right|^{2} +\frac{\bar{I}_{k,1}^{d}}{\sigma^{2}} + \frac{\bar{I}_{k,2}^{d}}{\sigma^{2}} \right) \right] \\ &\overset{(c)}{\leq} \textmd{E}\left[\log_{2} \left(1 + \frac{\bar{I}_{k,1}^{d}}{\sigma^{2}} + \frac{\bar{I}_{k,2}^{d}}{\sigma^{2}} \right)\right] \\ &\overset{(d)}{\leq} \log_{2} \left(1 + \textmd{E} \left[\frac{\bar{I}_{k,1}^{d}}{\sigma^{2}}\right] + \textmd{E} \left[\frac{\bar{I}_{k,2}^{d}}{\sigma^{2}}\right]\right) \end{aligned}  $$

Where (a) comes from the fact that $(\mathbf {u}_{k}^{d},\mathbf {v}_{k}^{d})$ is designed based on the perfect CSI, and $(\bar {\mathbf {u}}_{k}^{d},\bar {\mathbf {v}}_{k}^{d})$ is designed according the quantized CSI by the same IA scheme, (b) follows from the fact that $\bar {I}_{k,1}^{d} > 0, \bar {I}_{k,2}^{d} > 0$ and log2(·) is a monotonically increasing function, (c) follows the fact that **H**_*k*,*k*_ and $\bar {\mathbf {H}}_{k,k}$ are independently distributed, (d) uses Jensen’s inequality.

This bound explains that the rate loss increases logarithmically with the sum of residual interference. To minimize the rate loss due to the quantization error, we must reduce the residual interference at each receiver as much as possible.

For inter-user interference of the *d*th stream of the *k*th receiver, we can rewrite (9) as

13 $${} \begin{aligned} \frac{\bar{I}_{k,2}^{d}}{\sigma^{2}} &= \frac{P}{D\sigma^{2}}\sum\limits_{i=1,i \neq k}^{K} \sum\limits_{j=1}^{D} \left(\eta_{k,i}\left| (\bar{\mathbf{u}}_{k}^{d})^{H} \mathbf{H}_{k,i} \bar{\mathbf{v}}_{i}^{j} \right|^{2} \right)\\ &= \frac{P}{D\sigma^{2}}\sum\limits_{i=1,i \neq k}^{K} \sum\limits_{j=1}^{D} \left(\eta_{k,i}\left| (\bar{\mathbf{u}}_{k}^{d})^{H} \left((\cos \theta_{k,i})\bar{\mathbf{H}}_{k,i}\right.\right.\right. \\&\quad\left.\left.\left.+ (\sin \theta_{k,i}\Delta \mathbf{H}_{k,i})\right) \bar{\mathbf{v}}_{i}^{j} \right|^{2} \right)\\ &\overset{(e)}{=} \frac{P}{D\sigma^{2}}\sum\limits_{i=1,i \neq k}^{K} \sum\limits_{j=1}^{D} \left(\eta_{k,i}\sin^{2} \theta_{k,i} \left| (\bar{\mathbf{u}}_{k}^{d})^{H}\Delta \mathbf{H}_{k,i} \bar{\mathbf{v}}_{i}^{j} \right|^{2} \right)\\ &\overset{(f)}{=} \frac{P}{D\sigma^{2}}\sum\limits_{i=1,i \neq k}^{K} \sum\limits_{j=1}^{D} \left(\eta_{k,i}\sin^{2} \theta_{k,i} \left|{\vphantom{\left(\left| \mathbf{u}_{k}^{d} (\mathbf{v}_{i}^{j})^{H} \right) \right|}} vec(\Delta \mathbf{H}_{k,i})^{H} vec\right.\right.\\ &\quad\left.\left.\times\left(\mathbf{u}_{k}^{d} (\mathbf{v}_{i}^{j})^{H} \right) \right|^{2} \right) \end{aligned}  $$

Where (e) comes from the fact of (11), (f) follows the fact that $(\bar {\mathbf {u}}_{k}^{d},\bar {\mathbf {v}}_{i}^{j})$ is designed based on quantized channel matrix $\bar {\mathbf {H}}_{k,i}$ with IA approach. Defining $e_{k,i}^{d,j} = vec \left (\mathbf {u}_{k}^{d} (\mathbf {v}_{i}^{j})^{H} \right)$, we have

14 $$ \begin{aligned} \left\| e_{k,i}^{d,j} \right\|_{\textmd{F}}^{2} &= \left\| \mathbf{u}_{k}^{d} (\mathbf{v}_{i}^{j})^{H} \right\|_{\textmd{F}}^{2} = tr \left(\mathbf{u}_{k}^{d} (\mathbf{v}_{i}^{j})^{H} \mathbf{v}_{i}^{j} (\mathbf{u}_{k}^{d})^{H} \right)\\ &= tr \left(\mathbf{u}_{k}^{d} (\mathbf{u}_{k}^{d})^{H} \right) = \left\| \mathbf{u}_{k}^{d} \right\|_{\textmd{F}}^{2} = 1 \end{aligned}  $$

Thus, we can rewrite (14) as

15 $$ {}\frac{\bar{I}_{k,2}^{d}}{\sigma^{2}} = \frac{P}{D\sigma^{2}}\sum\limits_{i=1,i \neq k}^{K} \sum\limits_{j=1}^{D} \left(\eta_{k,i}\sin^{2} \theta_{k,i} \left| vec(\Delta \mathbf{H}_{k,i})^{H} e_{k,i}^{d,j}\right|^{2} \right)  $$

∀*k*≠*i*, noting that for the perfect IA we have $(\bar {\mathbf {u}}_{k}^{d})^{H} \bar {\mathbf {H}}_{k,i} \bar {\mathbf {v}}_{i}^{j} = 0$. It is equal to $vec \left (\bar {\mathbf {H}}_{k,i} \right)^{H} e_{k,i}^{d,j} = 0, e_{k,i}^{d,j}$ is an isotropic unit vector on the (*N*^2^−1) dimensional hyperplane orthogonal to $vec \left (\bar {\mathbf {H}}_{k,i} \right)^{H} $. Moreover, *vec*(**H**_*k*,*i*_)^*H*^ is the quantization distortion distributed in the same (*N*^2^−1)-dimensional null space of $vec \left (\bar {\mathbf {H}}_{k,i} \right)^{H} $. Therefore, the quantity $\left | vec(\Delta \mathbf {H}_{k,i})^{H} e_{k,i}^{d,j}\right |$ is beta-distributed with parameters (1,*N*^2^−2), and its expectation is equal to 1/(*N*^2^−1).

Since the random variables sin2*θ*_*k*,*i*_ and $\left | vec(\Delta \mathbf {H}_{k,i})^{H} e_{k,i}^{d,j}\right |$ are independent, the upper bound of the expected inter-user interference of the *d*th stream of the *k*th receiver can be obtained as

16 $$ \begin{aligned} {}\textmd{E}\left[ \frac{\bar{I}_{k,2}^{d}}{\sigma^{2}} \right] &\leq \frac{P}{D\sigma^{2}}\sum\limits_{i=1,i \neq k}^{K} \sum\limits_{j=1}^{D}\\ &\quad\times \left(\eta_{k,i} \textmd{E}\left[\sin^{2} \theta_{k,i}\right] \textmd{E}\left[\left| vec(\Delta \mathbf{H}_{k,i})^{H} e_{k,i}^{d,j}\right|^{2}\right] \right) \end{aligned}  $$

From [23], the expectation of sin2*θ*_*k*,*i*_ is given by $\Gamma (N^{2}) 2^{-B_{k,i}/(N^{2}-1)}\phantom {\dot {i}\!}$, where *B*_*k*,*i*_ denotes the number of feedback bits for link from transmitter *i* to receiver $j, \bar {\Gamma }(N^{2}) = \Gamma (1/(N^{2}-1))/(N^{2}-1)\phantom {\dot {i}\!}$, and *Γ*(*x*) represents the gamma function of *x*. Substituting above results into (16), we have

17 $$ \begin{aligned} \textmd{E}\left[ \frac{\bar{I}_{k,2}^{d}}{\sigma^{2}} \right] &\leq \frac{P}{D\sigma^{2}}\sum\limits_{i=1,i \neq k}^{K} \sum\limits_{j=1}^{D} \left(\eta_{k,i} \frac{\bar{\Gamma}(N^{2})}{N^{2}-1} 2^{-\frac{B_{k,i}}{N^{2}-1}}\right)\\ &= \frac{P}{\sigma^{2}}\sum\limits_{i=1,i \neq k}^{K} \left(\eta_{k,i} \frac{\bar{\Gamma}(N^{2})}{N^{2}-1} 2^{-\frac{B_{k,i}}{N^{2}-1}}\right) \end{aligned}  $$

Similarly, we have

18 $$ \begin{aligned} \textmd{E}\left[ \frac{\bar{I}_{k,1}^{d}}{\sigma^{2}} \right] \leq \frac{P(D-1)}{D\sigma^{2}} \eta_{k,i} \frac{\bar{\Gamma}(N^{2})}{N^{2}-1} 2^{-\frac{B_{k,k}}{N^{2}-1}} \end{aligned}  $$

Therefore, the expectation of sum interference to noise ratio for the *d*th stream of *k*th receiver is given by

19 $$ \textmd{E}\left[ \frac{{I_{k}^{d}}}{\sigma^{2}} \right] = \textmd{E}\left[ \frac{I_{k,1}^{d}}{\sigma^{2}} \right] + \textmd{E}\left[ \frac{I_{k,2}^{d}}{\sigma^{2}} \right] \leq \sum\limits_{i=1}^{K}\left(a_{k,i} \frac{P}{\sigma^{2}} 2^{-\frac{B_{k,i}}{N^{2}-1}}\right)  $$

Where {*a*_*k*,*i*_} in (19) is defined as

20 $$ a_{k,i}=\left\{ \begin{array}{l} \frac{D-1}{D}\eta_{k,i}\frac{\bar{\Gamma}(N^{2})}{N^{2}-1}, i = k \\ \eta_{k,i}\frac{\bar{\Gamma}(N^{2})}{N^{2}-1}, i \neq k \end{array}\right.  $$

From (12), we have

21 $$ \Delta {R_{k}^{d}} \leq \log_{2} \left(1 + \sum\limits_{i=1}^{K}\left(a_{k,i} \frac{P}{\sigma^{2}}2^{-\frac{B_{k,i}}{N^{2}-1}} \right) \right)  $$

Thus, the rate loss of the *k*th receiver can be written as

22 $$ \begin{aligned} \Delta R_{k} &= \sum\limits_{d=1}^{D} \Delta {R_{k}^{d}} \leq D\log_{2} \left(1 + \sum\limits_{i=1}^{K}\left(a_{k,i} \frac{P}{\sigma^{2}}2^{-\frac{B_{k,i}}{N^{2}-1}} \right) \right) \\ & = \Delta R_{k}^{upper} \end{aligned}  $$

### Feedback-bits allocation

As shown in (22), given transmit power, antenna numbers and feedback bits, we can obtain the upper bound of throughput loss. Hence, the result is useful for the design, analysis, and optimizations of the MIMO interference channels. It is obvious that allocating the same amount feedback bits for all links may degrade the rate of users. Motivated by the above observations, we consider that the different links might have different fading gain with heterogeneous path loss in this subsection to minimize the upper-bound of sum leakage interference and improve the throughput for each receiver. Based on the throughput loss analysis in the previous subsection, we perform dynamic feedback-bits allocation at each receiver in order to minimize the rate loss of each user by exploiting the heterogeneity of path loss. Moreover, the feedback-bits that minimize the sum residual interference also minimize the rate loss of *k*th user since the log2(·) is monotonically increasing. Specifically, we need to determine the optimal feedback-bits to minimize the sum residual interference of each receiver using the following optimization.

#### **Problem 1**

Dynamic feedback-bits allocation for the *k*th receiver

23 $$ \min_{\Psi_{k}} \sum\limits_{i=1}^{K} \left(a_{k,i} 2^{-\frac{B_{k,i}}{N^{2}-1}} \right)  $$

24 $$ s.t. \sum\limits_{i=1}^{K} B_{k,i} \leq B_{f}  $$

Where *Ψ*_*k*_={*B*_*k*,1_,⋯,*B*_*k*,*K*_}, and *B*_*k*,1_,⋯,*B*_*k*,*K*_ are non-negative integers.

In order to solve the problem 1, we formulate the lagrangian with multiplier *γ*, and take derivative with respect to *B*_*k*,*i*_ and *γ*. Then, we get

25 $$ L = \sum_{i=1}^{K} \left(a_{k,i}2^{-\frac{B_{k,i}}{N^{2}-1}} \right) + \gamma \left(\sum_{i=1}^{K} B_{k,i} -B_{f} \right)  $$

and

26 $$ \frac{\partial L}{\partial B_{k,i}} = -\frac{a_{k,i}\ln 2}{N^{2}-1}2^{-\frac{B_{k,i}}{N^{2}-1}} + \gamma = 0  $$

27 $$ \frac{\partial L}{\partial \gamma} = \sum_{i=1}^{K}B_{k,i} - B_{f} = 0  $$

From (26), we can get

28 $$ B_{k,i} = (N^{2}-1)\left(\log_{2} \left(\frac{a_{k,i}}{N^{2}-1} \right) + b \right)  $$

Where *b*= log2(ln2/*γ*). Combine (28) and (24) with *B*_*k*,*i*_≥0. The number of optimal feedback-bits as the optimal solution to problem 1 is obtained as

29 $$ B_{k,i} = (N^{2}-1)\left(\log_{2} \left(\frac{a_{k,i}}{N^{2}-1} \right) + \hat{b} \right)  $$

Where $\hat {b}$ satisfies $\sum _{k=1}^{K}B_{k,i} = B_{k}$, and [*x*]^+^ implies that [*x*]^+^= max(*x*,0). the solution of (29) could be found through the famous waterfilling algorithm, to avoid repetition, it is not described in this paper. For more details, one may refer to [17]. It is noteworthy that the number of feedback-bits should be integer, then the optimal feedback-bits are determined as the nearest integer to them.

## Simulation results

In this section, we evaluate the performance of the proposed scheme in term of the throughput by simulation. We focus on a three-hexagonal, multi-cell layout while each hexagonal containing a transmitter near its center, and a receiver locate the edge of cell. The distance between transmitter and its desired receiver distribute randomly between 0.8∗*r* and *r*, where *r* is the radius of inscribed circle of hexagon and is assumed to be 400 m. We evaluate the sum rate by mean of a Monte-Carlo simulation (averaging over 1000 realizations) with random generated small-scale fading **H**_*k*,*i*_ and the location of receivers. The path loss is determined by the model shown in (2) and the noise power is given by -113 dBm. We compare the mean sum rates obtained using three different CSI feedback schemes, perfect feedback, equal feedback and proposed dynamic feedback, as a function of the transmit power for the cases *K*=3,*N*=2,*d*=1;*K*=3,*N*=4,*d*=2 and *K*=3,*N*=4,*d*=2, respectively. The total number of feedback-bits for each receiver is set to 20.

**Fig. 2:**
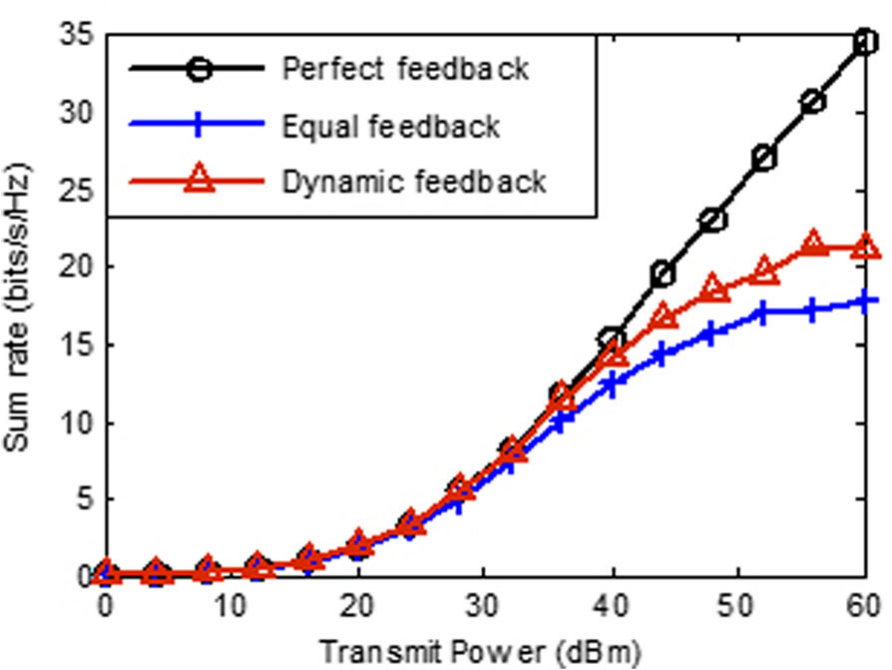
Average sum rate vs transmit power with *N*=2,*d*=1,*K*=3

**Fig. 3:**
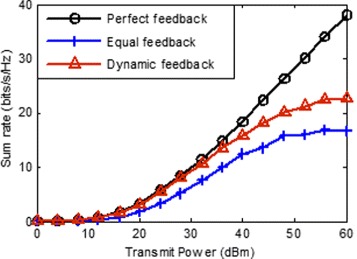
Average sum rate vs transmit power with *N*=4,*d*=1,*K*=3

First, we compare the sum of average rate of three CSI feedback schemes with *K*=3,*N*=2,*d*=1. As seen in Fig. 2, the sum rate loss is small when the transmit power is lower than 40 dBm, this is because that the sum residual interference is too small to degrade the rate of receivers. However, when the transmit power is larger than 40 dBm, the throughput loss due to the limited feedback is significant. It is shown that our proposed dynamic feedback-bits allocation scheme have decided advantage over the equal feedback-bits allocation. This is because of that the sum residual interference is reduced by our proposed dynamic feedback-bits allocation scheme, and that increase with transmit power.

secondly, we evaluate the sum of average rate with *K*=3,*N*=4,*d*=1. As seen in Fig. 3, the sum rate loss introduced by equal feedback is significant when the transmit power is large than 15 dBm. However, the performance of proposed dynamic feedback is near with the perfect feedback scheme. The difference is that the interference is more complex than last simulation, the benefit of the proposed dynamic feedback scheme is larger than last simulation. it is obviously that the average sum rate is also limited by the strong interference when the transmit power is larger than 40 dBm.

**Fig. 4:**
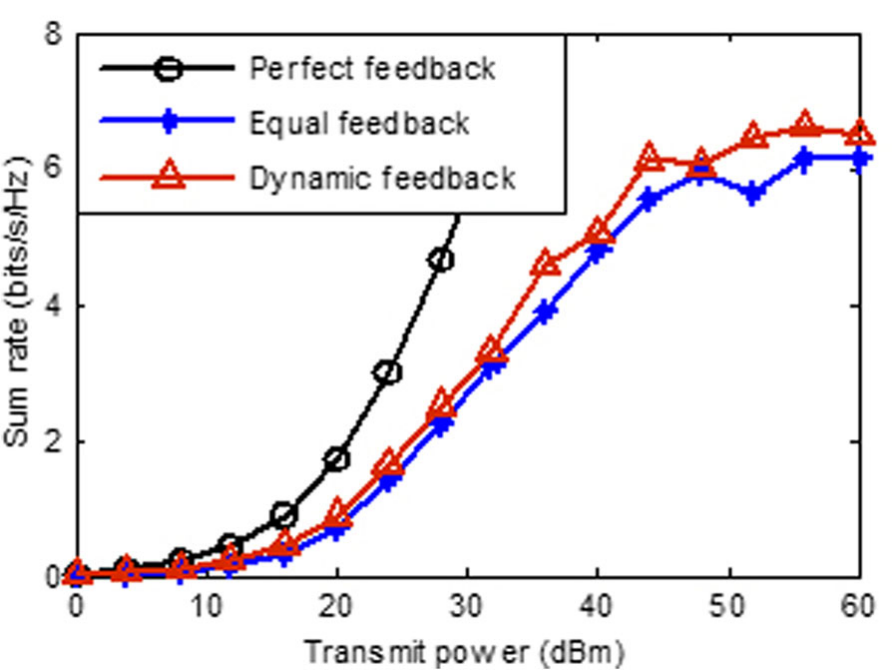
Average sum rate vs transmit power with *N*=4,*d*=2,*K*=3

Finally, we investigate the performance of three CSI feedback schemes. It is obvious that the performance in Fig. 4 is much less than in Figs. 2 and 3, this is because the intra-user residual interference is much more than inter-user residual interference when dof is more than 1, and that reduces the system performance seriously. Hence, it is important for each user to select the optimal number of degree of freedom from the perspective of performance. This problem will be investigated in my future works.

## Conclusions

In this paper, we consider MIMO interference channel with heterogeneous path loss and derive the performance of IA under limited feedback. Using the derived upper bound, we proposed a dynamic feedback bit allocation scheme to adapt to the path loss. We analyze the performance bounds under the proposed dynamic feedback scheme, in terms of the transmit power. Simulation results show that the proposed scheme enhance feedback efficiency.
